# Striking the Right Note: Assessing the Recipient for Liver Transplantation in Low- and Middle-Income Countries

**DOI:** 10.5152/TJAR.2021.1459

**Published:** 2022-02-01

**Authors:** Muhammad Irfan Ul Haq, Malika Hameed, Bruce Duncan

**Affiliations:** 1 Department of Anaesthesiology, Aga Khan University Hospital, Karachi, Pakistan; 2 Department of Anaesthesiology, St James’s University Hospital, Leeds, United Kingdom

**Keywords:** Anaesthesiologists, end-stage liver disease, liver, liver transplantation, living donors, preoperative care, organ transplantation

## Abstract

More than 50 years have passed since Starzl et al. did the first liver transplant. Since then the transplant speciality has witnessed enormous growth and at present more than 1 000 000 liver transplants have been performed to date in over 100 liver transplant centers around the world. In Europe and North America, the predominant mode is deceased donor liver transplantation, while in Turkey and most of the Asian countries, the living donor liver transplant or split liver transplantation is the most widely available method for liver transplantation. The etiology of end-stage liver disease is also different in developed and developing countries.

Liver recipients usually have multiple comorbidities and in addition, derangements in liver functions also indirectly affect other systems. The anaesthesiologist plays a very crucial role as a perioperative physician concerning liver transplantation. He is the lead person involved, from preoperative workup to intraoperative management and postoperative care in critical care units. Anaesthesiologists are also actively involved in developing organ transplant pathways and protocols for perioperative assessments.

Although there are local protocols and pathways for assessing liver transplant recipients, there is a lack of standardization in the literature for such assessments. This article highlights essential aspects in assessing liver transplant recipients and the role of some specific assessment tools and establishes a standardized protocol for selecting and optimizing suitable patients, thereby reducing the mortality and morbidity associated with this major surgery.

**Main Points**

- The burden of liver diseases is enormous in low- and middle-income countries. Subsequently, the incidence of liver failure is also on the rise.
- Liver recipients usually have multiple comorbidities, and derangements in liver functions also indirectly affect other systems.
- This article highlights essential aspects in assessing liver transplant recipients and the role of some specific assessment tools and establishes a standardized protocol for selecting and optimizing suitable patients, thereby reducing the mortality and morbidity associated with this major surgery.

## Introduction

More than 50 years had passed since the first liver transplant by Starzl et al.^1^ in 1963. He was unsuccessful in his first 5 liver transplant attempts as none of his patients survived for more than 23 days, mainly because of reperfusion injury, infection, coagulopathy, and graft rejection, leading to sepsis and liver failure. With some modifications in the immunosuppression regime and his surgical technique, he finally documented his first successful liver transplant in 1967 at the University of Colorado.^1^ At present, more than 1 000 000 liver transplants have been performed in over 100 liver transplant centers around the world. The 1-year survival rate in most of the advanced centers is more than 90% .^2^ This success accomplishment was not possible without the perseverance and hard work of some enthusiastic individuals in this field. The perpetual desire to innovate and find novel approaches to overcome hurdles is the driving force that made it possible to pass that million liver transplant landmark. 

Better organizational setup, coordinated teamwork between different subspecialties, selection of suitable candidates, and availability of logistics are the key elements in developing a successful program. Most of the centers across Europe and the USA developed an organ transplant system based on a central organ donor bank, transplant coordinators, organ retrieval teams, and protocols to streamline the smooth functioning of the transplant services. In Europe and North America, the predominant mode is deceased donor liver transplantation (DDLT), while in Turkey and most Asian countries, the living donor liver transplant (LDLT) or split liver transplantation is the most widely available method for liver transplantation.^2,3^

A multidisciplinary team should consist of a hepatobiliary surgeon, transplant anaesthesiologist, and hepatologist as permanent members. Supporting subspecialties include radiology, histopathology, oncology, hematology, and nephrology. 

Liver recipients usually have multiple comorbidities, and in addition, derangements in liver functions also indirectly affect other systems. On the other hand, living liver donors usually fall under the classification of American Society of Anaesthesiologists I or II. The anaesthesiologist plays a very crucial role as a perioperative physician concerning liver transplantation. He is the lead person involved from preoperative workup to intraoperative management and postoperative care in critical care units. Anaesthesiologists are also actively involved in developing organ transplant pathways and protocols for perioperative assessments.

The burden of liver diseases is enormous in low- and middle-income countries. Subsequently, the incidence of liver failure is also on the rise. In contrast to high-income countries where alcoholic liver disease, drug overdose, and autoimmune hepatitis are the leading causes of liver failure, hepatitis (B and C) is the significant cause of cirrhosis burden in low-income countries.^4^

### Clinical and Research Consequences

Although there are local protocols and pathways for assessing liver transplant recipients, there is a lack of standardization in the literature for such assessments. This article highlights essential aspects in assessing liver transplant recipients and the role of some specific assessment tools and establishes a standardized protocol for selecting and optimizing suitable patients, thereby reducing the mortality and morbidity associated with this major surgery.

### Assessment of a Recipient for Liver Transplantation

The assessment process can be divided into 3 stages:

- Confirmation of the diagnosis of chronic liver disease.
- Assessment of generalized health status with a particular focus on cardiovascular, respiratory, and central nervous systems. Exclude any absolute contraindications for the transplantation procedure and optimize any relative contraindications at this stage.
- The extent of severity, risk stratification, and family counseling about possible outcomes and complications that may arise immediately or later in the course.

## A. Establishing the Diagnosis

Most of the time patients come to the preoperative assessment clinic with a confirmed diagnosis. It is pertinent to review all the previous laboratory investigations related to the diagnosis. It will avoid the repetition of investigations and unnecessary delays. Recipient workup will be similar for cadaveric and living donor procedures. Following first-line investigations are recommended in a recipient (Table 1).

### Histology and Radiology

Liver biopsy is the gold standard in establishing the diagnosis of chronic liver disease, but it has its own demerits. Ultrasound-guided fine-needle biopsy is an invasive procedure that carries the risk of infection, bleeding, bile leak, and injury to adjacent organs. Because of the heterogeneous nature of parenchymal damage, multiple samples are required. In contrast, the ultrasound abdomen is noninvasive, reliable, and conventionally acceptable to the patients as a first approach. It provides early information on liver morphological appearance and portal hypertension status and can reveal liver hemodynamics with Doppler flow studies. Computed tomography and MRI can further aid in the diagnosis, but they are expensive and may require contrast injections as well. Computed tomography is especially helpful in the assessment of assessing hepatic artery, hepatic vein, and portal venous circulation status.^5^

Routine oesophagal endoscopy is advisable to rule out grade II and III varices before the transplant procedure. 

## B. Assessing Silent Comorbidities

### Evaluation of Cardiovascular System–Role of CPET

Liver transplantation is a major prolonged surgery involving significant physiological changes and a high level of surgical stress. Patients coming for liver transplantation have a significant functional limitations. Therefore, it is quite pertinent to assess the functional status of the heart and quantify morbidity and mortality accordingly. Major transplant centers have adopted cardiopulmonary exercise testing (CPET) across Europe. A national survey conducted by Reeves et al.^6^ in 2018 showed that CPET services continued to expand and it is now doubled since 2011; more than 30 000 tests are performed annually for patients having major abdominal surgeries.^6^ Two systematic reviews and more than 35 cohort studies support the use of CPET to evaluate the functional reserves and predict pre- and post-procedure complications in major surgeries.^7,8^

### Evaluation of Respiratory System: Role of Pulmonary Function Test

The incidence of pulmonary dysfunction is not uncommon in cirrhotic patients waiting for liver transplantation. Alves et al.^9^ reported hypoxia in half of the patients with advanced liver disease. The etiology is multifactorial and can be divided into 3 broad categories: gas exchange abnormalities (hepatopulmonary syndrome), portopulmonary hypertension, and hepatic hydrothorax.^10^ Pulmonary dysfunction has a significant impact on postoperative morbidity, especially concerning ventilator days and length of hospital stay.^11^ Therefore, it is relevant to rule out underlying pulmonary functions in an otherwise normal-looking recipient waiting for liver transplantation. 

### Evaluation of Nutritional Status: Role of Handgrip Testing

Nutritional status is directly related to pre- and postoperative complications in patients with end-stage liver disease. Multiple factors are responsible for this malnutrition and associated sarcopenia.^12^ There are different methods for evaluation of nutritional status like body mass index (BMI) and subjective global assessment (SGA). Anthropometric measurements, bioelectrical impedance analysis, and dual energy x-ray absorptiometry are used to assess the degree of sarcopenia. These tests have some limitations of their own; for instance, poor inter-observer agreement with SGA: generalized body edema and ascites will invariably affect BMI.^13^

Like muscle mass, muscle strength has been proposed for predicting the degree of sarcopenia. Handgrip strength (HGS) has been found to correlate well with muscle strength. It is a simple and valid method used in cirrhotic patients and can be measured using a dynamometer in a non-dominant hand.^14^ In hepatic encephalopathy, ammonia is converted to glutamine in the skeletal muscles, subsequently affecting the HGS. Lower values of HGS have been associated with a longer length of stay in an intensive care unit (ICU) and a higher likelihood of postoperative infections in transplant patients.^14^ It is interesting to note that no correlation was found between grip strength and hepatorenal syndrome, variceal bleeding, and spontaneous bacterial peritonitis, but age and smoking were associated with reduced grip strength in cirrhotic patients.^12^

## C. Risk Stratification: Role of Scoring Systems

Liver transplantation is the gold standard therapeutic procedure for patients with end-stage liver disease. With advancement and success in liver transplant procedures, there comes an imbalance between the availability of deceased donor organs and the number of recipients waiting for that organ. Different programs were initiated to overcome this burden, including encouragement of living donors, encouragement of deceased organ donation programs, and development of multiple scoring systems to quantify the risk of perioperative mortality and prioritize the patients on the waiting list for liver transplantation. Before 2002, patients with end-stage liver disease were classified according to the severity of their disease as super urgent, urgent, semi-urgent, and in elective categories for the liver transplantation, based on the severity of their disease. The Child–Turcotte–Pugh score was the only available scoring system for assessing the severity of the end-stage liver disease. Since then many scoring systems have been developed to predict mortality and prioritize the waiting list according to the severity of the disease. 

Child–Turcotte–Pugh Score: 

It was developed in 1973 to predict surgical outcomes in patients with sophageal varices. Since then this score has undergone modifications and is currently used to assess the severity and prognosis of chronic liver disease and cirrhosis. The severity of liver disease in this scoring system is determined on 5 clinical features: total bilirubin level, serum albumin, the international normalized ratio for prothrombin time (INR), degree of ascites, and the grade of hepatic encephalopathy. There are a few drawbacks to this scoring system. For instance, the degree of ascites and encephalopathy are subjective and depend upon physicians’ judgment as well as are affected by the use of lactulose.^15^

Model for End-Stage Liver Disease (MELD):

It was initially developed at the Mayo clinic in patients undergoing transjugular intrahepatic portosystemic shunt procedures. The Model for End-Stage Liver Disease (MELD) is a numerical scale ranging from 6 (less ill) to 40 (gravely ill) used for patients aged 12 and above. The derived score is based on a formula that used 3 laboratory parameters: serum total bilirubin, INR, and creatinine as per the equation below^16^: 

MELD = 3.78 × ln [serum bilirubin (mg dL-1)] + 11.2 × ln [INR] + 9.57 × ln [serum creatinine (mg dL-1)] + 6.43

The number derived from this scoring system can predict how urgently one needs a liver transplant within the next 3 months.^17^ If there is a history of renal dialysis twice a week, then the creatinine value is fixed at 4.0 mg dL-1. The Model for End-Stage Liver Disease scores are always reported as a whole number and any value less than 1 in the above equation is rounded to 1.0. The Model for End-Stage Liver Disease score of a patient is variable as it depends upon his or her current clinical condition and therefore the MELD score is routinely reassessed on multiple occasions while on the waiting list. 

As of January 2016, modification was made, and in 2016, serum sodium level was also included in MELD score and now its referred to as MELD-Plus or MELD-Na score. In some European countries, the age of the recipient is also added to MELD-Na. It is then referred to as *i*MELD.^18^

United Kingdom Model for End-stage Liver Disease (UKELD): 

The UK Transplant developed a new scoring system of their own in 2008. This incorporated serum bilirubin, creatinine, sodium, and INR. A score of 49 and above is the minimum score required to be added to the waiting list and this score predicts 1-year mortality of 9% without transplantation.^19^ UKELD was validated and showed no association with overall post-transplant survival, but there is a direct relationship with both the duration of stay in an ICU and overall hospital stay.^20^

## D. Counseling and Consenting

After getting all the laboratory investigations and assessments from different specialities involved in liver transplantation, a second appointment is scheduled with the patient and his close relatives. Ideally, in the presence of a hepatologist, anaesthesiologist, and hepatobiliary surgeon, a comprehensive discussion about the course of the diseases, its prognosis without transplantation, available alternatives, and morbidity and mortality associated with liver transplant should be done. Postoperative outcomes, including graft rejection, liver failure, and prolong intensive care stay, need to be discussed and documented. Any queries from patients and their immediate family members are addressed at this point. Risk stratification in 3 categories (low, intermediate, and high risk) is documented. All consents related to the transplant procedure are taken. These should include consent for invasive monitoring, massive blood and blood product transfusions, and use of cell salvage. 

## Conclusion

Liver transplantation is a challenging job that needs an organized setup and exemplary teamwork. A standardized approach toward the selection of the right candidate is essential. Most of the developing countries are still struggling to achieve the desired results. Availability of resources, expertise, and technical assistance from the international community are a few of the limiting factors in the development of liver transplant speciality in low- and middle-income countries. The coordination among regional liver transplant associations will help deal with the increasing numbers of potential recipients in a particular region. Anaesthetist plays a paramount role, from assessing and optimizing the recipients to postoperative critical care management. The development of programs for training in transplant speciality will definitely improve the outcome.

## Figures and Tables

**Table 1.** First-Line Investigations Recommended in a Recipient

Group	Tests	Comments
Hematology	Complete blood profile	
Septic Markers	Serum lactate CRP	
Coagulation	PT, aPTT, INR	Repeat them the day before surgery.
Liver function tests	Bilirubin, alkaline phosphatase, gamma GGT, AST, ALT, total protein, serum albumin, alpha-1 antitrypsin, and alpha fetoprotein	
Biochemistry	Serum Sodium, Potassium, Chloride, Bicarbonate, Magnesium and Phosphorus	Repeat them on the day of surgery.
Renal parameters	Urea, creatinine, creatinine clearance, urinalysis	eGFR is quite often low, but if there is a reason for it, like diuretic usage or mild hepatorenal syndrome, then there is no need to refer to a nephrologist. If patients require a combined liver-kidney transplant or may need dialysis after transplant, then nephrology referral is appropriate.
Metabolic	Fasting glucose, fasting lipid profile, vitamin D levels, bone density	If already done, there is no need to repeat lipid profile, vitamin D, and bone density.
Respiratory	Chest x-ray	
Heart	ECG/ECHO	Especially look for pulmonary artery pressure and if it is >30-35 mm Hg, refer to the cardiologist. Remember, echo estimations of PA pressure are often inaccurate. High PA pressure is not an absolute contraindication to the surgery but will worsen the patient’s risk assessment.
Microbiology	Cultures of urine, ascitic fluid, and sputum	Ideally, it should be done 1 week before the surgery.
Serology	HBV, HCV and HIV, CMV, EBV, and VZV	It depends upon the history and after taking informed consent.
Blood arrangements	Blood group, 6 units of packed red blood cells, 6 units of fresh frozen plasma and 6 units of cryoprecipitate	Ideally, arrangements should be made 1 week before the surgery date.

CRP, C-reactive protein; GFR, estimated glomerular filtration rate; GGT, gamma-glutamyl transferase, PA, pulmonary artery, PT, protrombin, aPTT, activated protrombin time, INR, international normalizing ratio, ECG, electrocardiography, ECHO, echocardiography.

## References

[1] Meirelles Júnior RF Salvalaggio P Rezende MB et al. Liver transplantation: history, outcomes and perspectives. Einstein (Sao Paulo). 2015;13(1):149 152. 10.1590/S1679-45082015RW3164) 25993082 PMC4977591

[2] Akbulut S Yilmaz S . Liver transplantation in Turkey: historical review and future perspectives. Transplant Rev (Orlando). 2015;29(3):161 167. 10.1016/j.trre.2014.12.002) 25535023

[3] Narasimhan G Living donor liver transplantation in India. Hepatobiliary Surg Nutr. 2016;5(2):127 132. 10.3978/j.issn.2304-3881.2015.09.01) 27115006 PMC4824736

[4] Sepanlou SG, Safiri S, Bisignano C, et al . The global, regional, and national burden of cirrhosis by cause in 195 countries and territories, 1990-2017: a systematic analysis for the Global Burden of Disease Study 2017. Lancet Gastroenterol Hepatol. 2020;5(3):245 266. 10.1016/S2468-1253(19)30349-8) 31981519 PMC7026710

[5] Yeom SK Lee CH Cha SH Park CM . Prediction of liver cirrhosis, using diagnostic imaging tools. World J Hepatol. 2015;7(17):2069 2079. 10.4254/wjh.v7.i17.2069) 26301049 PMC4539400

[6] Reeves T Bates S Sharp T et al. Cardiopulmonary exercise testing (CPET) in the United Kingdom—a national survey of the structure, conduct, interpretation and funding. Perioper Med (Lond). 2018;7:2. 10.1186/s13741-017-0082-3) 29423173 PMC5787286

[7] Moran J Wilson F Guinan E McCormick P Hussey J Moriarty J . Role of cardiopulmonary exercise testing as a risk assessment method in patients undergoing intra-abdominal surgery: a systematic review. Br J Anaesth. 2016;116(2):177 191. 10.1093/bja/aev454) 26787788

[8] Older PO Levett DZH . Cardiopulmonary exercise testing and surgery. Ann Am Thorac Soc. 2017;14(Supplement_1):S74 S83. 10.1513/AnnalsATS.201610-780FR) 28511024

[9] Alves L Sant’Anna CC March Mde F et al. Preoperative pulmonary assessment of children for liver transplantation. Pediatr Transplant. 2008;12(5):536 540. 10.1111/j.1399-3046.2007.00845.x) 18194351

[10] Bozbas SS Eyuboglu F . Evaluation of liver transplant candidates: a pulmonary perspective. Ann Thorac Med. 2011;6(3):109 114. 10.4103/1817-1737.82436). 21760840 PMC3131751

[11] Kia L Cuttica MJ Yang A et al. The utility of pulmonary function testing in predicting outcomes following liver transplantation. Liver Transpl. 2016;22(6):805 811. 10.1002/lt.24426) 26929108

[12] Dela Cruz AC Vilchez V Kim S et al. A prospective analysis of factors associated with decreased physical activity in patients with cirrhosis undergoing transplant evaluation. Clin Transplant. 2015;29(11):958 964. 10.1111/ctr.12602). 26263921 PMC4624476

[13] Ciocîrlan M Cazan AR Barbu M Mănuc M Diculescu M Ciocîrlan M . Subjective global assessment and handgrip strength as predictive factors in patients with liver cirrhosis. Gastroenterol Res Pract. 2017;2017:8348390. 10.1155/2017/8348390) 28804497 PMC5540494

[14] Fernandes SA Bassani L Nunes FF Aydos ME Alves AV Marroni CA . Nutritional assessment in patients with cirrhosis. Arq Gastroenterol. 2012;49(1):19 27. 10.1590/s0004-28032012000100005) 22481682

[15] Peng Y Qi X Guo X . Child–Pugh Versus Score for the assessment of prognosis in Liver Cirrhosis: A systematic review and meta-analysis of observational studies. Med (Baltim). 2016;95(8):e2877. 10.1097/MD.0000000000002877) PMC4779019 26937922

[16] Wiesner R Edwards E Freeman R et al. Model for end-stage liver disease (MELD) and allocation of donor livers. Gastroenterology. 2003;124(1):91 96. 10.1053/gast.2003.50016) 12512033

[17] Zhao H Gu X Zhao R Shi Y Sheng J . Evaluation of prognostic scoring systems in liver cirrhosis patients with bloodstream infection. Med (Baltim). 2017;96(50):e8844. 10.1097/MD.0000000000008844) PMC5815685 29390273

[18] Luca A Angermayr B Bertolini G et al. An integrated MELD model including serum sodium and age improves the prediction of early mortality in patients with cirrhosis. Liver Transpl. 2007;13(8):1174 1180. 10.1002/lt.21197) 17663415

[19] Luca A Angermayr B Bertolini G et al. An integrated MELD model including serum sodium and age improves the prediction of early mortality in patients with cirrhosis. Liver Transpl. 2007;13(8):1174 1180. 10.1002/lt.21197) 17663415

[20] Cholongitas E Burroughs AK . The evolution in the prioritization for liver transplantation. Ann Gastroenterol. 2012;25(1):6 13. 24713804 PMC3959341

[21] Barber K Madden S Allen J et al. Elective liver transplant list mortality: development of a United Kingdom end-stage liver disease score. Transplantation. 2011;92(4):469 476. 10.1097/TP.0b013e318225db4d) 21775931

